# Multiple cropping effectively increases soil bacterial diversity, community abundance and soil fertility of paddy fields

**DOI:** 10.1186/s12870-024-05386-w

**Published:** 2024-07-27

**Authors:** Haiying Tang, Ying Liu, Xiaoqi Yang, Guoqin Huang, Xiaogui Liang, Adnan Noor Shah, Muhammad Nawaz, Muhammad Umair Hassan, Alaa T. Qumsani, Sameer H. Qari

**Affiliations:** 1 School of Agriculture and Biotechnology, Hunan University of Humanities, Science and Technology, Loudi, 417000 China; 2 Key Laboratory of Crop Physiology, Ecology and Genetics Breeding, Jiangxi Agricultural University, Ministry of Education, Nanchang, China; 3 Research Center on Ecological Sciences, Jiangxi Agricultural University, Nanchang, 330045 China; 4 Department of Agricultural Engineering, Khwaja Fareed University of Engineering and Information Technology, Rahim Yar Khan 64200, Punjab Pakistan; 5 Department of Biology, Al-Jumum University College, Umm Al-Qura University, Makkah, Saudi Arabia

**Keywords:** Multiple winter cropping, Bacterial diversity, Bacterial community abundance, Soil fertility

## Abstract

**Background:**

Crop diversification is considered as an imperative approach for synchronizing the plant nutrient demands and soil nutrient availability. Taking two or more crops from the same field in one year is considered as multiple cropping. It improves the diversity and abundance of soil microbes, thereby improving the growth and yield of crops. Therefore, the present study was conducted to explore the effects of different multiple winter cropping on soil microbial communities in paddy fields. In this study, eight rice cropping patterns from two multiple cropping systems with three different winter crops, including Chinese milk vetch (CMV), rape, and wheat were selected. The effects of different multiple winter cropping on soil microbial abundance, community structure, and diversity in paddy fields were studied by 16 S rRNA high-throughput sequencing and real-time fluorescence quantitative polymerase chain reaction (PCR).

**Results:**

The results showed that different multiple winter cropping increased the operational taxonomic units (OTUs), species richness, and community richness index of the bacterial community in 0 ~ 20 cm soil layer. Moreover, soil physical and chemical properties of different multiple cropping patterns also affected the diversity and abundance of microbial bacterial communities. The multiple cropping increased soil potassium and nitrogen content, which significantly affected the diversity and abundance of bacterial communities, and it also increased the overall paddy yield. Moreover, different winter cropping changed the population distribution of microorganisms, and *Proteobacteria*, *Acidobacteria*,* Nitrospira*,* and Chloroflexi* were identified as the most dominant groups. Multiple winter cropping, especially rape-early rice-late rice (TR) andChinese milk vetch- early rice-late rice (TC) enhanced the abundance of *Proteobacteria*,* Acidobacteria*, and *Actinobacteria* and decreased the relative abundance of *Verrucomicrobia* and *Euryarchaeota*.

**Conclusion:**

In conclusion, winter cropping of Chinese milk vetch and rape were beneficial to improve the soil fertility, bacteria diversity, abundance and rice yield.

**Supplementary Information:**

The online version contains supplementary material available at 10.1186/s12870-024-05386-w.

## Introduction

Microbes have multiple metabolic functions in the farmland ecosystem, and they also participate in a large number of biochemical reactions [1, 2], which plays an important role in plant growth, soil carbon and nitrogen cycles [3, 4]. Microbial communities play a vital role in soil fertility, soil environment and optimization of ecological processes [2, 5]. However, community structure and diversity of microbes are affected by plant species, soil composition and quality. Microbial communities can respond sensitively to environmental changes and they are considered as indicators reflecting soil environmental changes [6]. Different factors, such as fertilization [4, 5], farming practices [7], cultivation methods [8] and land use methods [9, 10] affect soil microbial community.

The introduction of agro-ecological practices, including crop diversification, is considered as an important way to increase nutrient cycling, crop production, microbial activity and control pests and diseases [11, 12]. The diversification of cropping systems affect the soil microbes owing to differences in soil disturbance, substrate quality and quantity [12]. For instance, cropping diversity increases diversity of crop residues, which are different in chemical composition; therefore, this diversification supports great diversity and density of soil microbes [13]. Moreover, different plant species vary in their root architecture, and they also recruit specific communities of microbes by producing root exudates and signaling molecules, which increase the overall microbial community and can have a legacy impact on subsequent crops [14–16]. Thus, all these factors can interact with rotation-mediated effects on the soil microbes [17].

Intensifying cropping rotation provides a more stable environment as compared to fallow rotations due to continuous cover cropping, which promotes the abundance and diversity of soil bacteria and fungi [13, 18]. In diversified cropping, different straws are returned to the soil, which can also increase the diversity and abundance of soil microbes [19, 20]. The changes induced by straw returning in soil physical and chemical properties also drive changes in the microbial community which affect the overall microbial community structure and metabolic function [21–23].

The middle reaches of the Yangtze River is an important grain base commodity in China, and it is also a typical triple cropping and double cropping rice production area in China. Multi-cropping systems in paddy fields have been formed, which consist of the double cropping farming system including winter cropping (wheat/rape-single rice or ratooning rice) and the triple farming systems (winter green manure/wheat/rape-early rice-late rice) [24]. The change in rural labor force structure, the decline of comparative agricultural benefits, and the increase in production costs changed the planting structure in this region. As a result, the cropping system in this region has been changed from double rice to single rice resulting in a continuous increase of winter fallow field area [25]. These changes in planting structure inevitably affect the soil properties of paddy fields, thus affecting the diversity of soil microorganisms and the stability of microbial community structure in paddy fields.

Different studies have reported that winter green manures provide carbon and nitrogen sources for microbes which improve the richness of soil bacterial community and microbial diversity in paddy fields [26–29]. However, the application of different winter mulching crop straw induces significant changes in the diversity index of soil microbes. For instance, at the maturity stage of early rice, maximum Richness, Shannon, and McIntosh Indices were recorded with potato-double rice treatment followed by ryegrass-double rice, Chinese milk vetch(CMV) -double rice, and rape-double rice and lowest Richness, Shannon, and McIntosh Indices were reported in winter fallow [30].

The triple cropping pattern (rape-double rice rotation mode) significantly improved the soil microbial richness index but had no significant impact on the evenness and dominance index [31]. Nonetheless, most of the studies focused on the impact of single cropping mode on soil bacterial community structure, and limited reports are available about the effect of multiple winter cropping practices on soil properties, microbial diversity, and community structure. Therefore, based on six-year continuous field experiments, this study was conducted with the following objectives: (1) to explore the difference in the impact of different winter cropping on soil microbial diversity and community composition, soil physical and chemical properties, (2) to clarify the relationship between soil microbial community and soil physical and chemical properties to provide a theoretical basis for the rational application of winter multiple cropping cultivation techniques in the rice growing areas of middle reaches of the Yangtze River.

## Materials and methods

### Experimental site

The long-term positioning experiment was performed from October 2014 to December 2020 at Wannian Agricultural Science Research Institute, Jiangxi Province. The experimental site has an average annual temperature of 18.6℃, 1906 mm annual rainfall, and a sunshine duration of 1662 h. The experiment soil was red clay (0 ~ 20 cm) with a pH of 6.62, soil organic matter (SOC) 35.62 g·kg^− 1^, total nitrogen (TN), 2.22 g·kg^− 1^, alkali-hydrolyzable nitrogen(AN) 157.5 mg·kg^− 1^, total phosphorus (TP) 0.64·kg^− 1^, available phosphorus (AP) 13.44 mg kg^− 1^, total potassium (TK) 16.22 g kg^− 1^, available potassium (AK) 71.98 mg·kg^− 1^ and C/N ratio 9.31.

### Experimental treatments

The positioning experiment was conducted after the harvest of late rice in October 2014. A single-factor randomized block design was adopted, and two multiple cropping systems were set up, including three different winter crops Chinese milk vetch (CMV), rape, and wheat. In total, we set eight different treatments, including DN: winter fallow-middle rice; DC: Chinese milk vetch-middle rice; DR: rape- middle rice; DW: wheat- middle rice; TN: winter fallow-early rice-late rice; TC: Chinese milk vetch-early rice-late rice; TR: rape-early rice-late rice; and TW: wheat-early rice-late rice (Table 1). A 1.5 m wide protective belt was set around the plots, and each treatment was comprised of three replications.

**Table 1:** Details of experimental treatments used in study

Cropping system	Pattern of multiple cropping	Abbreviation	Straw incorporation
TCS	Chinese milk vetch-early rice-late rice	TC	All Chinese milk vetch and early rice straw were returned to the field; all late rice straw was returned as mulching
	rape-early rice-late rice	TR	All rape and early rice straw were returned to the field;15 cm late rice straw stubble was returned to the field
	wheat-early rice-late rice	TW	All wheat and early rice straw were returned to the field;15 cm late rice straw stubble was returned to the field
	Winter fallow-early rice-late rice	TN	All early rice straw was returned to field; 15 cm late rice straw stubble was returned to the field
DCS	Chinese milk vetch-middle rice	DC	All Chinese milk vetch straw was returned to the field and all middle rice straw was returned as mulching.
	Rape-middle rice	DR	All rape straw was returned to field; 15 cm middle rice straw stubble was returned to the field
	Wheat-middle rice	DW	All wheat straw was returned to field, and 15 cm middle rice straw stubble was returned to the field
	Winter fallow-middle rice	DN	15 cm rice straw stubble was returned to the field

*Note:* DCS and TCS mean double cropping system and triple cropping system, individually. In order to compare the effects of winter crops on a certain cropping system, in the comparative analysis, the winter fallow-middle rice model (DN) was used as the control of double winter multiple cropping system including TC, DR, DW, and the winter fallow-double cropping rice (TN) was used as the control of triple winter multiple cropping system including TC, TR, TW.

In the middle reaches of the Yangtze River in China. Double cropping rice (early rice and late rice) is a common planting pattern. Early rice is usually transplanted at the end of April and harvested in early July. After the early rice is harvested, the late rice is continued to be planted in the field, and the late rice is usually harvested in the middle or late October. Middle rice is usually transplanted at the beginning of May and harvested in the middle of August

### Experimental materials and fertilizer application

The compound fertilizers Sanyuan (N: P_2_O_5_: K_2_O = 15%: 15%: 15%) was applied to rape and wheat as base fertilizers, while urea was also applied to winter rape and wheat at the rates of 375 and 450 kg ha^− 1^, respectively. The amount of CMV straw returning to the field was 22,500 kg ha^− 1^, and the straw of CMV contained 0.36% N, 0.11% P_2_O_5_, and 0.28% K_2_O respectively. On the other hand, the amount of rape straw returned to the field was 19,500 kg ha^− 1^, and straw of rape contained 0.368% N, 0.15% P_2_O_5_, and 0.51% K_2_O, while the amount of wheat straw returning to the field was 10,500 kg ha^− 1^, and its straw contained 0.53% N, 0.13% P_2_O_5_, and 0.32% K_2_O respectively. The information regarding sowing and harvesting dates and varieties used in winter crops is given in Table 2, while information regarding rice varieties and sowing and harvesting dates is given in Table 3.

**Table 2:** Details of sowing and harvesting dates and varieties used in winter crop planting

Winter crops	Year	Variety	Sowing date	Harvest date	Planting pattern
Chinese milk vetch	2019	Yujiang wide leaves	2018/10/5	2019/5/3	broadcast
	2020	Yujiang wide leave	2019/10/1	2020/5/2	broadcast
Rape	2019	Ganyouza No.8	2018/11/5	2019/5/2	hole seeding
	2020	Ganyouza No.8	2019/10/28	2020/4/29	hole seeding
Wheat	2019	Yangmai 23	2018/10/31	2019/5/3	drill seeding
	2020	Yangmai 23	2020/10/28	2020/5/3	drill seeding

**Table 3:** Details of sowing and harvesting dates and varieties of ear and late rice

Rice season	Year	Variety	Transplanting date	Tillering stage	Booting stage	Full heading stage	Harvest date
Early rice	2019	Zhongzao 37	2019/5/5	2019/5/15	2019/6/10	2019/6/18	2019/7/15
	2020	Zhongzao 37	2020/5/8	2020/5/17	2020/6/10	2020/6/19	2020/7/14
Middle rice	2019	Jingliangyouhuazhan	2019/6/13	2019/6/20	2019/8/12	2019/8/18	2019/9/18
	2020	Jingliangyouhuazhan	2020/6/13	2020/6/22	2020/8/11	2020/8/18	2020/9/26
Late rice	2019	Rongyou huazhan	2019/7/20	2019/7/25	2019/8/27	2019/9/6	2019/10/14
	2020	Rongyou huazhan	2020/7/17	2020/7/25	2020/8/30	2020/9/10	2020/10/24

The chemical fertilizers were applied in the form of urea (N 46%), superphosphate (P_2_O_5_ 12%), and potassium chloride (K_2_O 60%) to fulfill the NPK requirements of rice crop. The amount of urea applied to early, middle, and late rice was 153.33 kg ha^− 1^, while superphosphate and potassium chloride was applied at the rate of 50.91 kg ha^− 1^ and 122.73 kg ha^− 1^ respectively, to fulfill P and K requirements of early, middle and late rice. All phosphate and potash fertilizers were applied as basal dose and nitrogen fertilizers were applied as basal fertilizer, tillering fertilizer (applied 5–7 days after transplanting), and panicle fertilizer (when panicle length was reached 1–2 cm).

### Field management

The seed rate of CMV, rapeseed and wheat crops were 30 kg ha^− 1^, 22.5 kg ha^− 1^, and 30 kg ha^− 1^, respectively. In all winter crops, compound fertilizers (375 kg ha^− 1^) were applied as base fertilizers. However, fertilizers were not applied to CMV, while to rapeseed and wheat crops, fertilizers were applied at the rates of 225 kg ha^− 1^ and 330 kg ha^− 1^, respectively. Moreover, CMV straw was returned to the paddy field at full flowering stage, while straws of rape and wheat were incorporated into the field after harvesting both crops. Additionally, early rice was planted at a planting density of 19 cm × 23 cm, while in middle and late rice, a planting density of 23 cm × 23 cm was maintained.

### Determination of soil bacterial diversity, soil physical and chemical properties

After harvesting late rice in October 2019, 24 soil samples (3 replicates for each treatment) were taken from 0 to 20 cm soil layer according to the five-point sampling method. After that, the collected samples were mixed, and they were immediately frozen and brought back to the laboratory. After removing debris, soil samples were divided into different parts: a part of fresh soil was frozen at -80 °C for determination of bacterial diversity (tested by Beijing Nuohe Zhiyuan Technology Co., Ltd. for determination) and the remaining soil samples were screened after air drying to determine soil properties. Appropriate DNA samples qualified for quality detection were diluted with sterile water and used as templates for polymerase chain reaction (PCR) amplification using specific primers 314 F/806R for bacterial V3 and V4 regions [32]. The same mixture was monitored by agarose gel electrophoresis and recovered using a gel recovery kit (Qiagen). The library was constructed with a library-building assay kit, and the qualified library was tested by the Illumina Miseq sequence test platform. Moreover, FLASH v1.2.7 software was used to splice the number of double-ended reads of soil bacteria obtained by high-throughput sequencing, and then Qiimev1.9.1 software was used to process the final valid Tags results. With Up-arsev7.0.1001 software, the final effective data results with 97% similarity were aggregated to obtain an operational taxonomic unit (OTU). The α-diversity index, including Chao1 index, Shannon index, and species number, were calculated by Mothur polymerization results of OTU. The soil pH was measured with pH meter (1:5 soil: distilled water) and soil N was determined with Kjeldahl method, and soil P and K was determined by sodium bicarbonate extraction (spectrophotometry), and ammonium acetate extraction flame photometry methods. For determination of microbial biomass carbon (MBC); 10 g moist soil was taken in two sets; one set was fumigated with chloroform for 24 h and other set was not fumigated. Later, both set of soil was extracted by using 50 ml of K_2_SO_4_ (0.5 M) and filtered to obtain the extract and MBC concentration was measured with carbon analyzer. The organic matter contents was measured with potassium dichromate method, and alkali hydrolyzable N was measured with alkali N-proliferation method while ammonium and nitrate N was determined with KCl extraction method.

### Data analysis

The SPSS22.0 software was used to analyze the data and analysis of variance (ANOVA) and Duncan’s method (Ducan) were used to test the significant differences among soil microbial abundance, soil physicochemical and plant properties (α = 0.05). The principal coordinate analysis (PCoA) [33] and redundancy analysis (RDA) [34] were performed to determine the correlation between soil properties and soil microbial population composition. Lastly, figures were made by using Microsoft Excel 2019 and R Studio.

## Results

### Diversity analysis of soil microbial community

**Fig. 1:**
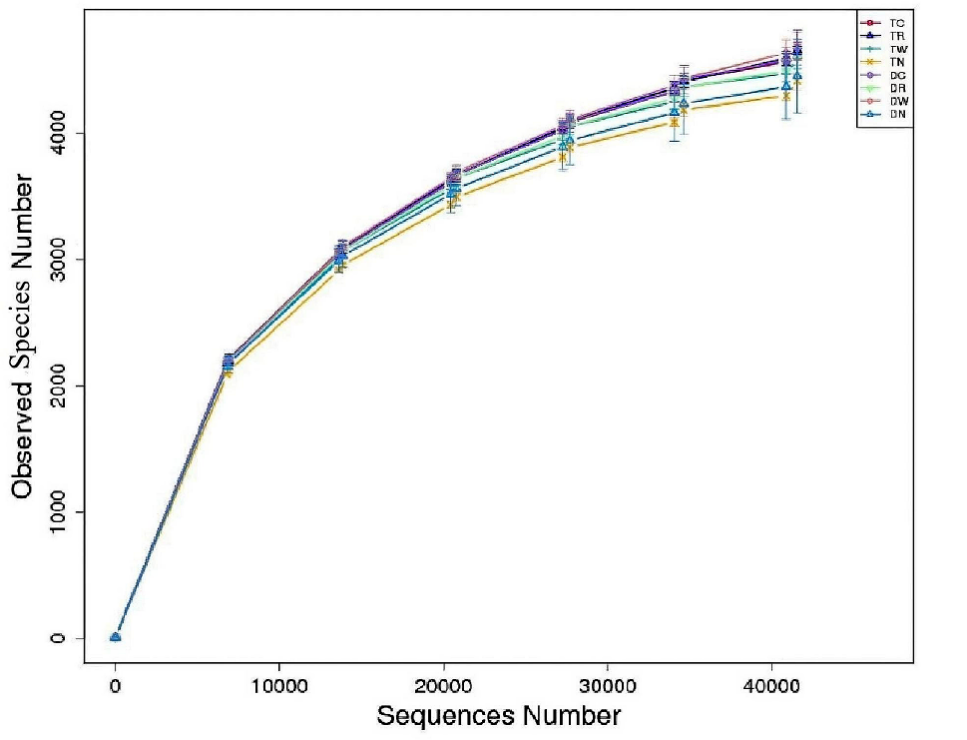
Rarefaction curve of bacteria OTUs abundance

The sample dilution curve (Fig. 1) is an important basis for characterizing the depth of high-throughput sequencing covering all microbial groups in the sample. The results showed that number of OTU in 24 soil samples rapid increased and slope of the dilution curve showed a downward trend and a slow increase over the time. Furthermore, the number of observed species plateaued at around 40,000 sequence number. This indicates that the sequencing depth was reasonable and the obtained data covered most of bacterial species in soil samples. The richness and diversity of microbial communities were calculated by using the OTU level method. The results indicate that the diversity and abundance index of microbial bacterial communities in paddy fields were significantly increased after six years of continuous multiple winter cropping.

**Fig. 2:**
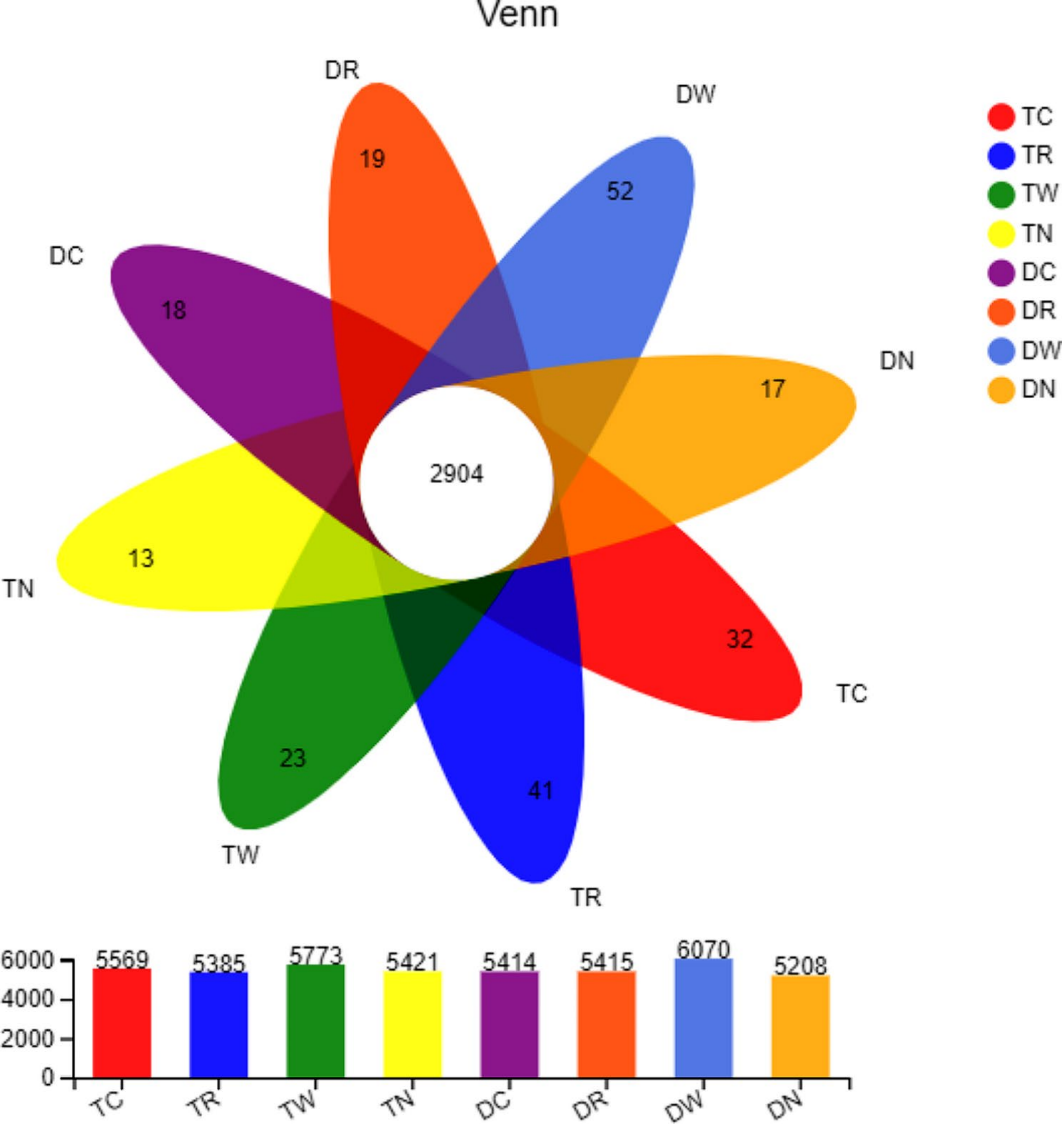
Venn figure of bacteria OTUs distribution. *Note* TC Chinese milk vetch-early rice-late rice; TR rape-early rice-late rice; TW wheat-early rice-late rice; TN Winter fallow-early rice-late rice; DC Chinese milk vetch-middle rice; DR Rape-middle rice; DW Wheat-middle rice; DN Winter fallow-middle rice

The average value of OTU in each treatment of the triple cropping system was 5575.67 and it was 2.85% higher than the control Fig. (2). The average value of OTU in each treatment of double cropping system was 5633. The number of OTUs in DC, DR and DW was 3.94%, 3.97% and 16.5% higher than control. The average Shannon index of each treatment in the triple cropping system was 10.62, which was 0.85% higher than that of the control. The treatment TC, TR and TW had 0.76%, 1.04% and 0.85% higher Shannon index than control. Moreover, average Shannon index of each treatment was 10.61, and the Shannon index of DC, DR and DW was 0.09%, 0.28% and 0.37% higher than DN. Furthermore, compared with double cropping system, the soil of triple cropping system had higher Shannon index, but the difference was not significant (*P* > 0.05).

The OTUs, species richness, Shannon index, and community richness index (Chao1 index, ACE index) of soil bacteria in TC, TR, TW, DC, DR, and DW were significantly higher than winter fallow treatments including DN and TN. The average Shannon index of each treatment in the triple cropping system was 10.62, which was 0.85% higher than control (Table 4). Treatments TC, TR, and TW had 0.76%, 1.04%, and 0.85% higher Shannon index than the control treatment, while the Shannon index of DC, DR, and DW was 0.09%, 0.28%, and 0.37% higher than DN treatment. The results indicate that triple winter cropping increased the community abundance index (Chao 1) of TC, TR, and TW by 8.78%, 12.04%, and 6.52% than TN. Similarly, triple cropping increased the ACE index of TC, TR, and TW by 6.35%, 6.29%, and 3%, respectively, as compared to TN (Table 4). The results indicate that double multiple winter cropping significantly increased the community abundance index Chao 1 and ACE in the paddy field. The results also indicate that the Chao 1 index of DC, DR, and DW was increased by 8.58%, 11.09%, and 11.58%, respectively, and the ACE index of aforementioned treatments was increased by 19.35%, 14.1%, and 19.35% respectively. The results also indicate that triple multiple winter cropping pattern had higher Chao 1 than double multiple winter cropping; however, double multiple winter cropping had higher ACE index.

**Table 4:** Bacterial community richness and diversity indices of different winter planting patterns

Cropping system	Treatment	Observed species	Coverage%	Diversity index		Richness index	
				Shannon	Simpson	Chao 1	ACE
TCS	TC	4653.33 ± 81.45a	0.97 ± 0.01a	10.61 ± 0.04a	0.99 ± 0.01a	5438.32 ± 130.29a	5813.07 ± 166.79a
	TR	4653.00 ± 43.35a	0.97 ± 0.01a	10.64 ± 0.04a	0.99 ± 0.01a	5601.18 ± 373.93a	5810.27 ± 214.27a
	TW	4559.67 ± 77.15ab	0.91 ± 0.01a	10.62 ± 0.04a	0.99 ± 0.01a	5325.18 ± 185.84ab	5630.17 ± 215.64a
	TN	4411.00 ± 82.61bc	0.97 ± 0.01a	10.53 ± 0.01a	0.98 ± 0.01a	4999.2 ± 65.61bc	5466.22 ± 318.08ab
DCS	DC	4578.67 ± 82.62ab	0.97 ± 0.01a	10.6 ± 0.09a	0.98 ± 0.01a	5252.38 ± 123.75ab	5912.13 ± 546.88a
	DR	4598.00 ± 82.63a	0.91 ± 0.01a	10.62 ± 0.07a	0.99 ± 0.01a	5373.81 ± 176.41a	5652.49 ± 140.61a
	DW	4685.00 ± 82.64a	0.97 ± 0.01a	10.63 ± 0.01a	0.99 ± 0.01a	5397.7 ± 111.92a	5897.36 ± 477.33a
	DN	4250.00 ± 82.65c	0.97 ± 0.01a	10.59 ± 0.08a	0.99 ± 0.01a	4837.46 ± 121.56c	4953.79 ± 159.39b
Average	DCS	4527.92 ± 71.14a	0.970 ± 0.01a	10.61 ± 0.03a	0.999 ± 0.01	5215.34 ± 203.01a	5603.94 ± 228.69a
	TCS	4569.25 ± 82.64a	0.970 ± 0.01a	10.60 ± 0.06a	0.999 ± 0.01	5340.97 ± 133.41a	5679.93 ± 331.05a

*Note* TC Chinese milk vetch-early rice-late rice; TR rape-early rice-late rice; TW wheat-early rice-late rice; TN Winter fallow-early rice-late rice; DC Chinese milk vetch-middle rice; DR Rape-middle rice; DW Wheat-middle rice; DN Winter fallow-middle rice

The results of Spearman rank correlation analysis (Table 5) showed that the soil physical and chemical properties of winter multiple cropping patterns had a significant impact on the diversity and abundance of bacterial communities. The observed species and Shannon index had a positive correlation with total soil potassium (TK) (*P* < 0.01), while Simpson index, ACE index, and goods coverage also had a positive correlation with TK (*P* < 0.05). Moreover, observed species showed a significant positive correlation with available nitrogen (AN) (*P* < 0.05). Thus, these results suggested that soil potassium and nitrogen are the important factors that can affect bacterial diversity and abundance in different winter cropping patterns.

**Table 5:** Correlation between bacterial community diversity and abundance index in different multiple winter cropping systems

	Observed species	Shannon	Simpson	Chao1	ACE	Goods coverage
pH	0.216	0.092	0.002	0.042	0.13	-0.069
SOM	-0.259	-0.232	-0.276	-0.07	-0.155	0.095
TN	-0.05	0.037	-0.211	-0.087	-0.109	0.159
TP	-0.333	-0.186	-0.097	-0.303	-0.352	0.338
TK	-0.542**	-0.564**	-0.417*	-0.397	-0.454*	0.408*
AN	-0.411*	-0.392	-0.262	-0.193	-0.251	0.179
AP	-0.135	0.092	0.253	-0.265	-0.246	0.265
AK	-0.386	-0.285	-0.21	-0.215	-0.287	0.218
SB	-0.226	-0.001	0.019	-0.031	-0.161	0.12
NO_**3**_^**−**^	0.02	-0.06	-0.15	0.061	0.06	-0.054
NH₄⁺	0.243	0.311	0.321	0.257	0.196	-0.192
MBC	0.126	0.258	0.278	0.219	0.155	-0.173
MBN	0.354	0.219	0.042	0.082	0.189	-0.103

*Note* SOM, Soil organic matter; TN, Total nitrogen; TP, Total phosphorus; TK, Total potassium; BD, Bulk density; AN, Available nitrogen; AP, Available phosphorus; AK, Available potassium; NO_3_^−^-N, nitrate nitrogen; NH_4_^+^-N, Ammonium nitrogen; MBC, Microbial biomass carbon; MBN, Microbial biomass nitrogen. * and ** in the table mean *p* < 0.05, *p* < 0.01, respectively

### Analysis of soil bacterial community structure and similarity

**Fig. 3:**
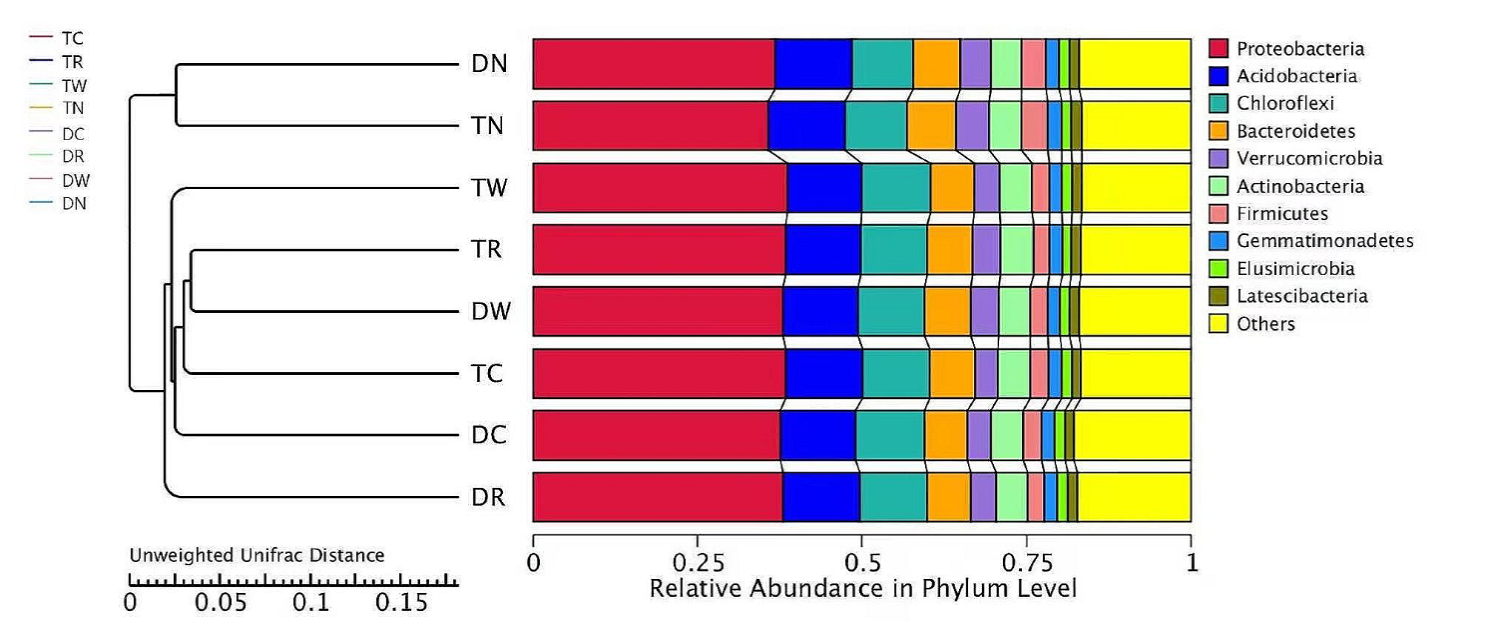
Composition relative abundance and UPGMA cluster analysis of soil microbial communities under different multiple winter cropping systems

The results indicate that at the bacteriophyta level, the similarity of bacterial community structure was higher among different winter multiple cropping treatments (Fig. 3). The first ten phyla with higher abundance were *Proteobacteria*,* Acidobacter*,* Nitrospira*,* Chloroflexi*,* Bacteroides*,* Actinobacteria*,* Verrucomicrobia*,* Firmicutes*,* Germatimonadetes*, and *Euroarcheota*. The relative content analysis of bacteria showed that the dominant groups were *Proteus*,* Acidobacter*,* Nitrothyrobacter*,* Campylobacter*,* Bacteroides*, and *Actinomyces*. The results of the current study showed that multiple winter cropping increased the abundance of *Proteus*,* Acidobacter*, and *Actinomyces* and decreased the abundance of *Verrucomycetes* and *Archaea*.

**Fig. 4:**
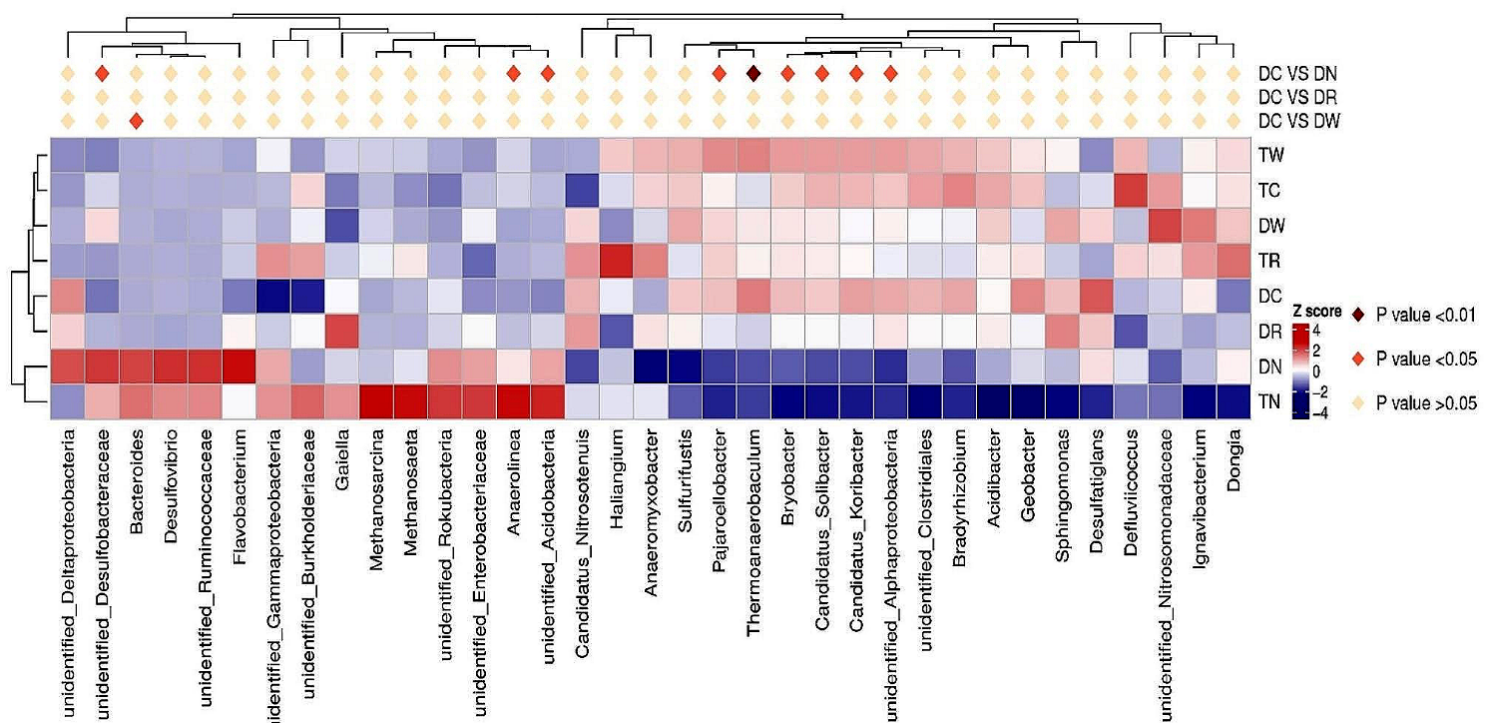
Heatmap analysis of soil bacterial community under different cropping patterns

The results indicate that six different multiple winter cropping patterns and two winter fallow treatments including DN and TN were located in two clusters, which indicates a significant difference among multiple winter cropping and winter fallow cropping (Figs. 3 and 4). The treatments DC and TC were located in the same cluster, which indicates that the same winter crop can have a similar microbial population under different cropping systems. Moreover, DR and TR with the same winter wheat were also found in the same cluster, which indicates that the same winter crop of rape planted under different cropping mode also had a similar microbial population. Nonetheless, TR and DR treatments with the same winter crop rape were noticed in different clusters, which shows that different cropping structures can change the microbial population.

**Fig. 5:**
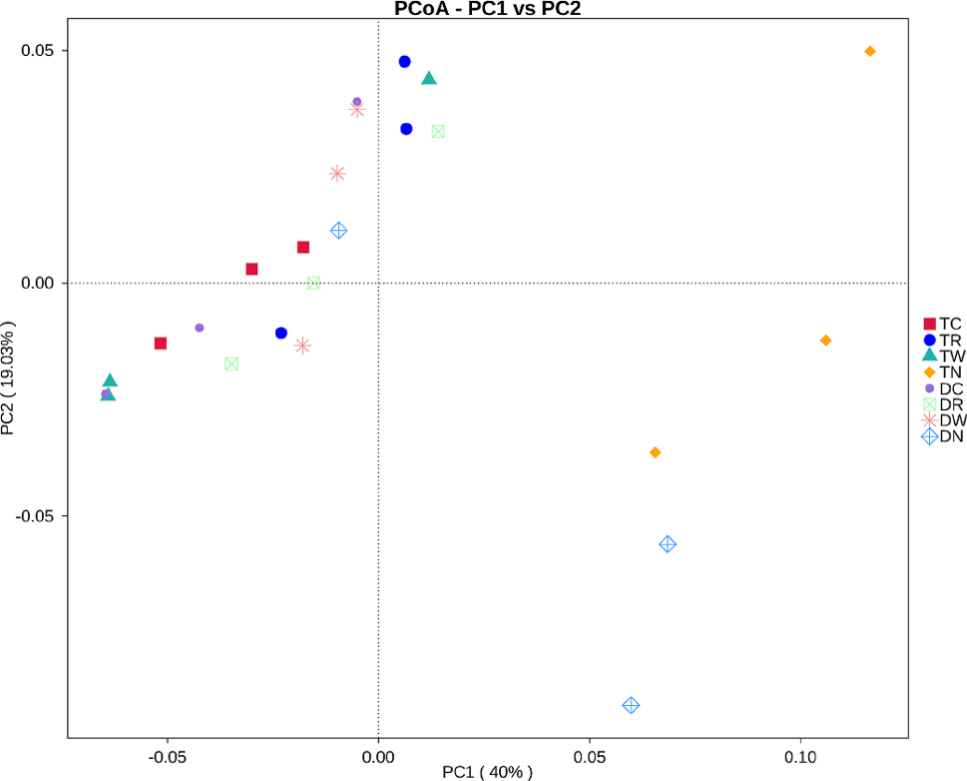
PCoA analysis based on weighted Unifrac distance

According to PCoA the distance between the repeated treatments was relatively close owing to the higher similarity of microbial community structure (Fig. 5). However, the structure of the soil bacterial community was different in different treatments. In particular, winter fallow treatments TN and DN were significantly different from other multiple winter cropping treatments. Moreover, 40% of bacterial community changes were explained by PC1 axis, and 19.03% of bacterial community changes were explained by PC2 axis (Fig. 5). The bacterial community showed differences due to the different winter multiple cropping patterns. Overall, winter fallow treatments TN and DN were relatively close; similarly, winter multiple cropping patterns were relatively close. This indicates that winter multiple cropping had a significant impact on microbial abundance and community structure.

### Relationship between soil microbial community structure and soil physical and chemical properties

The results indicate that the soil physical and chemical properties were significantly changed after six years of continuous winter cropping (Table 6). The results showed that different cropping systems effectively increased soil pH in winter by 5.58%~1.55%. Similarly, different winter cropping also had a significant impact on soil organic matter (SOM), and the concentration of SOM ranged between 27.45 ~ 35.1 g kg^− 1^. However, winter multiple cropping had a non-significant impact on soil TN and TP, though contents of TN and TP ranged between 2.03 ~ 2.4 g kg^− 1^ and 0.68 ~ 0.71 g kg^− 1^, respectively. The results showed that AN content of TC, TR, and TW was significantly different than the control treatment. The results showed that AN content of TC, TR, and TW were reduced by 22.04%, 23.43%, and 20.59%, respectively. On the other hand, each winter multiple cropping treatments significantly increased the soil AP 0.59% ~38.29% except DC treatment. Conversely, each multiple winter cropping reduced the AK concentration by 18.61%~44.57% except for the DR treatment.

**Table 6:** Soil physical and chemical properties under different winter multiple planting patterns

Cropping system	Treatm-ent	pH	SOM(g·kg^− 1^)	TN(g·kg^− 1^)	TP(g·kg^− 1^)	TK(g·kg^− 1^)	BD( g·cm^− 3^)	AN(mg·kg^− 1^)	AP(mg·kg^− 1^)	AK(mg·kg^− 1^)	NO_3_^−^-*N* (mg·kg^− 1^)	NH_4_^+^-*N* (mg·kg^− 1^)	MBC(mg·kg^− 1^)	MBN(mg·kg^− 1^)
TCS	TC	6.55 ± 0.1bc	27.45 ± 2.14d	2.39 ± 0.15a	0.7 ± 0.04a	12.53 ± 1.12abc	1.14 ± 0.06b	111.32 ± 2.1bc	26.67 ± 2.58b	39.17 ± 4.49b	0.07 ± 0.02c	1.26 ± 0.36bc	437.25 ± 39.92bc	40.19 ± 1.01de
	TR	6.69 ± 0.1ab	30.15 ± 0.2c	2.4 ± 0.01a	0.71 ± 0.18a	11.58 ± 1.57c	1.14 ± 0.01b	109.34 ± 4.21bc	31.06 ± 3.58a	27.67 ± 6.24c	0.11 ± 0.07bc	1.73 ± 0.26ab	509.04 ± 11.84a	59.23 ± 3.78a
	TW	6.7 ± 0.15ab	32.26 ± 1.39bc	2.39 ± 0.04a	0.68 ± 0.05a	12.14 ± 0.54bc	1.15 ± 0.13b	113.4 ± 8.4bc	26.31 ± 1.74bc	26.68 ± 7.54c	0.14 ± 0.01bc	1.51 ± 0.09abc	425.06 ± 37.64bc	46.95 ± 1.44bcd
	TN	6.58 ± 0.03bc	35.1 ± 1.56a	2.16 ± 0.51a	0.69 ± 0.05a	13.28 ± 0.25ab	1.11 ± 0.02b	142.8 ± 8.4a	22.46 ± 2.24 cd	48.13 ± 1.52a	0.15 ± 0.07bc	1.27 ± 0.56bc	418.54 ± 21.71bc	52.14 ± 2.32abc
DCS	DC	6.81 ± 0.12a	32.25 ± 0.38bc	2.38 ± 0.08a	0.63 ± 0.17a	11.78 ± 2.18bc	1.2 ± 0.09b	103.6 ± 4.85c	19.22 ± 2.79d	26.67 ± 1.72c	0.12 ± 0.02bc	1.94 ± 0.41a	462.55 ± 10.43b	51.62 ± 7.64abc
	DR	6.61 ± 0.06abc	34.05 ± 1.21ab	2.44 ± 0.13a	0.68 ± 0.06a	13.12 ± 0.77abc	1.18 ± 0.04b	119.65 ± 3.72b	28.09 ± 3.09ab	50.64 ± 1.72a	0.17 ± 0.06ab	0.14 ± 0.02d	424.25 ± 6.44bc	55.93 ± 5.58ab
	DW	6.61 ± 0.11abc	33.74 ± 0.37ab	2.03 ± 0.68a	0.71 ± 0.05a	12.59 ± 0.48abc	1.17 ± 0.04b	103.6 ± 6.42c	26.14 ± 0.84bc	36.18 ± 1.5b	0.19 ± 0.03ab	1.04 ± 0.19c	461.34 ± 14.24bc	45.19 ± 5.44cde
	DN	6.45 ± 0.14c	32.42 ± 0.82bc	2.38 ± 0.04a	0.7 ± 0.03a	14.01 ± 0.58a	1.37 ± 0.04a	116.2 ± 6.42b	25.73 ± 2.43bc	46.65 ± 3a	0.25 ± 0.08a	1.3 ± 0.17bc	416.65 ± 12.03c	36.51 ± 6.75e

*Note* SOM: Soil organic matter; TN: Total nitrogen; TP: Total phosphorus; TK: Total potassium; BD: Bulk density; AN: Available nitrogen; AP: Available phosphorus; AK: Available potassium; NO_3_^−^-N: nitrate nitrogen; NH_4_^+^-N: Ammonium nitrogen; MBC: Microbial biomass carbon; MBN: Microbial biomass nitrogen

The findings of our study indicate that multiple cropping reduced the soil nitrate nitrogen (NO_3_-N) as compared to winter fallow. The results indicate that triple cropping system has soil NO_3_-N 35.62% lower than the double cropping system. The average NO_3_-N content of the triple cropping system was 28.89% lower than TN treatment, while the average NO_3_-N content of the double cropping system was 36% lower than DN treatment (Table 6).

The results also indicate that multiple winter cropping significantly increased MBC content. The results showed that the MBC content of TC, TR, and TW in each triple multiple winter cropping treatment was higher by 4.47%, 21.66%, and 1.55% than the TN treatment (Table 6). On the other hand, MBC contents of DC, DR, and DW in the double winter cropping system were higher by 11.02%, 1.82%, and 10.73% than DN. Moreover, the microbial biomass nitrogen (MBN) of the double cropping system was 4.35% higher than winter cropping system. Likewise, in the triple multiple cropping system, the MBN content of TR was higher by 13.6% as compared winter fallow treatment. Additionally, MBN was also increased in each multiple cropping mode, and MBN of DC, DR, and DW treatments had 41.39%, 53.19%, and 23.77% higher MBN than DN treatment (Table 6).

### Correlation between soil bacterial community and soil physical and chemical properties under different cropping patterns

**Fig. 6:**
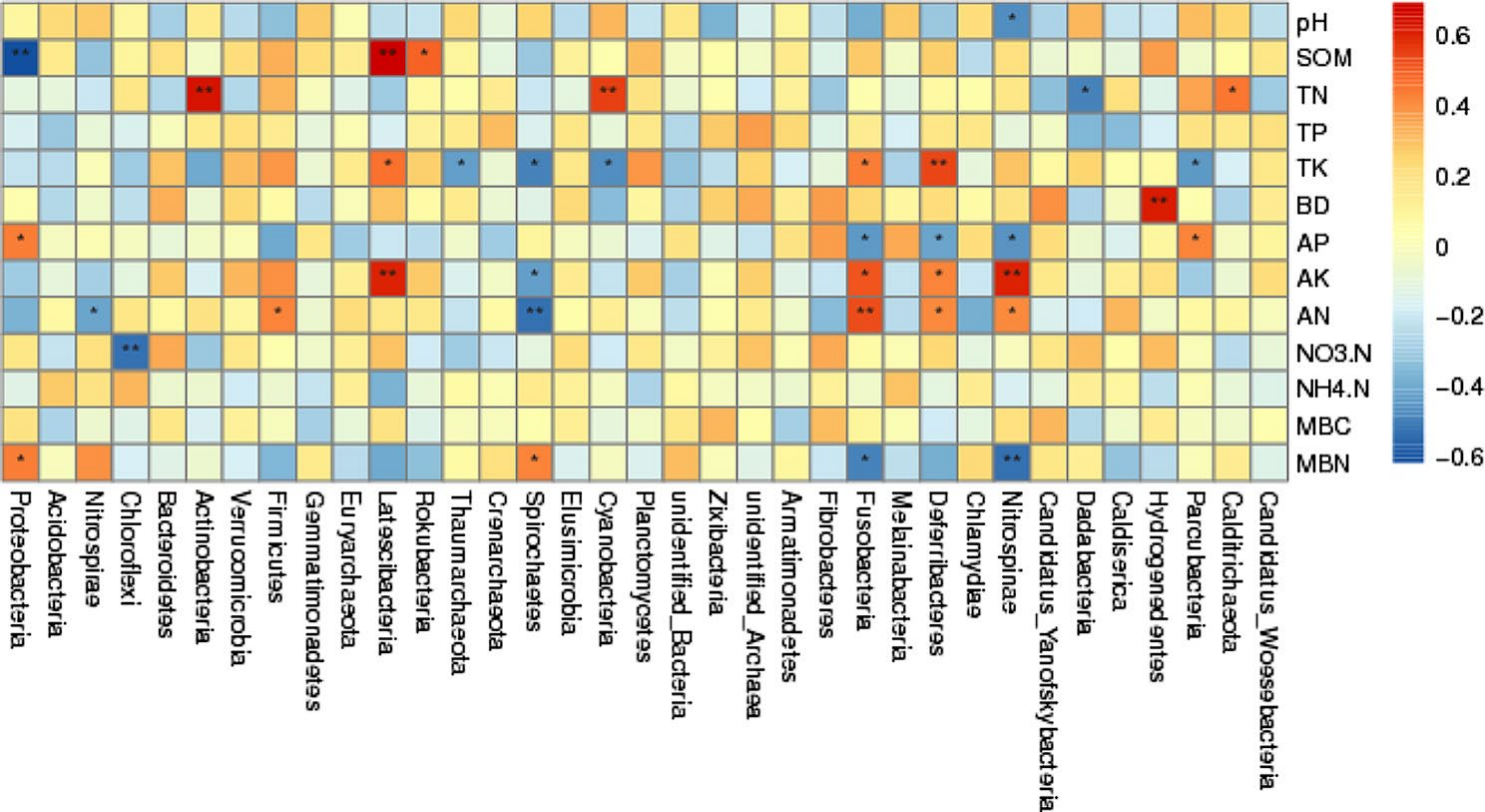
Correlation coefficients between soil microbial community composition (phyla) and soil physiochemical properties. *Note* * and ** in the fig. mean *p* < 0.05, *p* < 0.01, respectively

Spearman rank correlation analysis (Fig. 6) indicates that soil physical and chemical properties significantly affected the composition of the microbial community. The results indicate that the relative abundance of *Proteobacteria* was negatively correlated with the SOM (*P* < 0.01); however, it was positively correlated with AP and MBN (*P* < 0.05). On the other hand, *Nitrospirae* also had a negative correlation with AN, *Chloroflexi* was negatively correlated with NO_3_-N, while, *Actinobacteria* and *Latescibacteria* had a positive correlation with TN. Likewise, *Latescibacteria* showed a significant positive correlation with SOM, AK, and TK. There was also a significant positive correlation between *Rokubacteria* and SOM; however, *Spirochaetes* had a negative correlation with TK, AK, and AN while it had a positive correlation with MBN.

**Fig. 7:**
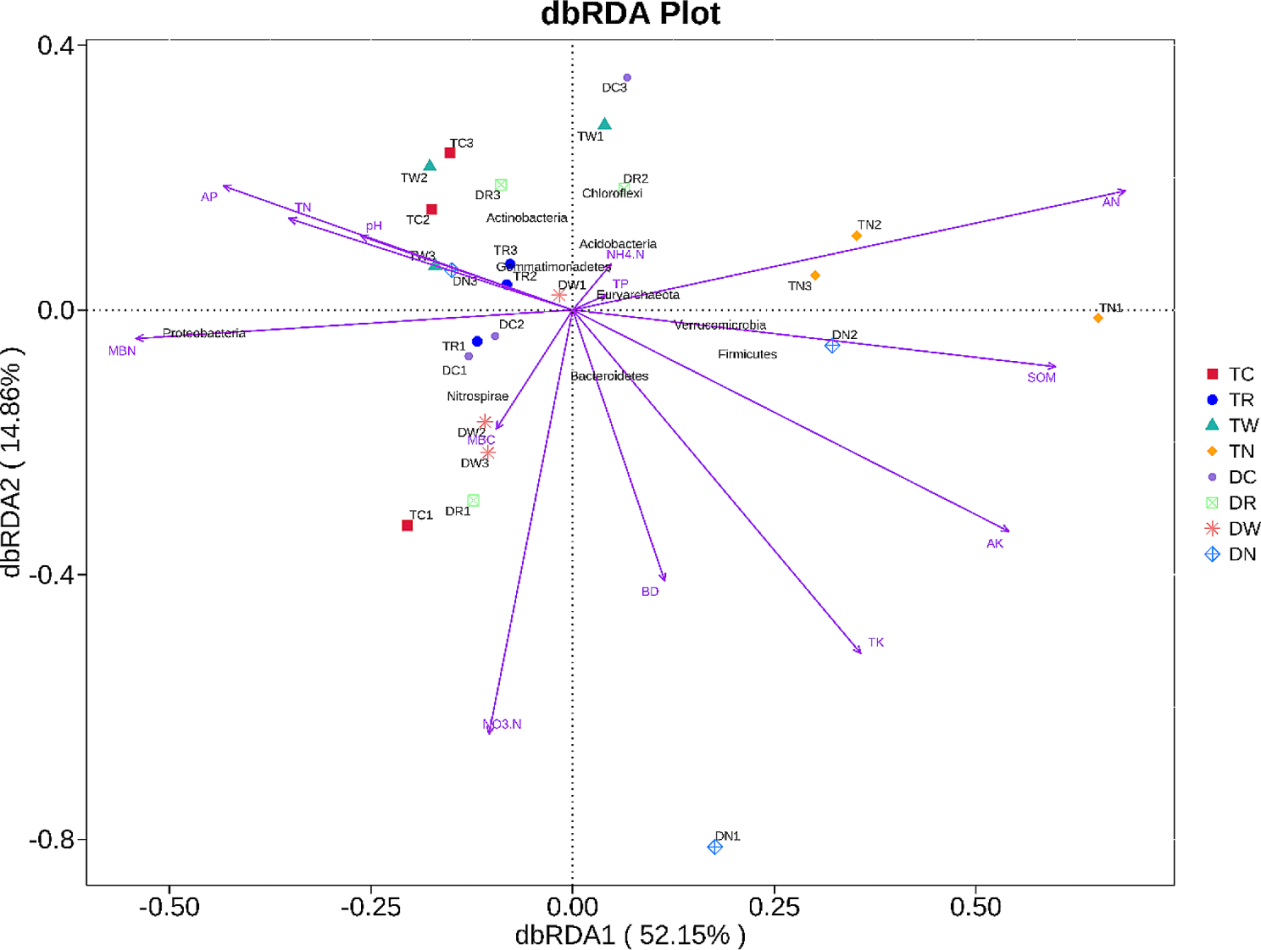
Distance-based redundancy analysis of soil bacterial community and soil chemical properties under different winter multiple cropping system

Similarly, *Cyanobacteria* showed a positive association with TN and a negative correlation with TK, and *Fusobacteria* and *Deferribacteres* showed a positive link with TK, AK, AN, and a negative correlation with AP. On the other hand, *Nitrospinae* showed a positive association with AK and AN and a negative correlation with pH, AP, and MBN. Moreover, there was a negative correlation between *Dadabateria* and TN and positive relation between *Hydrogenedentes* and BD. Lastly, *Paracubacteria* had a positive correlation with TK and a negative correlation with AP, while *Calditrichaeota* showed a negative correlation with TN. Thus, it is concluded that SOM, TN, TK, AP, AK, ANA, MBN, BD, and pH are the main factors affecting the composition of microbial communities. In addition, the Monte Carlo permutation test was used to analyze the relation between soil properties and bacterial community composition. The results indicate that soil pH, AK, and MBN were the main factors affecting bacterial communities. These results are supported by redundancy analysis (RDA) with the first (RDA1) and second (RDA2) showed 52.15% and 14.86% of the variation in overall bacterial community composition (Fig. 7). The RDA analysis also showed that pH, AK, and MBN were the most important factors that significantly affected the microbial community composition as compared to other factors (Fig. 7).

### Effect of different cropping modes on rice yield

The results indicate that the annual yield of triple cropping system (TCS) was higher than double rice cropping system (Table 7). The results showed that in 2019, the average yield of TCS was 67.5% higher than the DCS. Furthermore, in TCS, the annual yield of TC, TR, and TW was 1.99%, 15.64%, and 14.58% higher than control treatment (Table 4). In the case of DCS, the annual production of DC, DR, and DW was 3.23%, 18.84%, and 20.13% higher than the control treatment DN. The annual yield in 2020 also showed the same trend, and the average yield of the TCS was 55.66% higher than that of the DCS. In TCS, the annual yield of TC, TR, and TW was 7.77%, 24.57%, and 26.58% higher than the control treatment. In DCS, the annual yield of DR and DW was 22.57% and 23.99% higher than the control treatment. Moreover, based on the annual yield of two years; the increase in the yield of each treatment in TCS with winter multiple cropping was 17.17%~20.58% than the winter fallow. Lastly, the yield of each treatment under DCS with winter multiple cropping was increased by 1.42%~22.06% than the winter fallow.

**Table 7:** Comparison of yield among different multiple rice cropping patterns

Year	Cropping system	Pattern	Winter crop	Early rice	Late rice	Annual yield
2019	TCS	TC	-	7762.96 ± 128.14ab	8424.54 ± 95.36c	16187.51 ± 222.33b
		TR	1401.23 ± 28.29b	7944.44 ± 66.77a	9007.82 ± 48.90a	18353.5 ± 104.02a
		TW	1709.88 ± 56.58a	7707.9 ± 61.34b	8768.13 ± 115.62b	18185.9 ± 75.69a
		TN	-	7585.95 ± 83.34b	8287.45 ± 44.79d	15871.4 ± 95.11c
	DCS	DC	-	9560.49 ± 62.08ab	-	9560.49 ± 62.08e
		DR	1228.4 ± 74.84c	9777.78 ± 155.21a	-	11006.18 ± 145.14d
		DW	1728.4 ± 65.03a	9397.58 ± 136.78bc	-	11125.97 ± 126.04d
		DN	-	9261.36 ± 91b	-	9261.36 ± 91f
2020	TCS	TC	-	6892.93 ± 132.97b	8175.95 ± 95.65a	15058.88 ± 168.87b
		TR	1166.67 ± 80.72b	7385.86 ± 99.65a	7734.63 ± 123.03b	16287.15 ± 280.81a
		TW	1524.69 ± 65.03a	7359.6 ± 135.19a	7764.48 ± 89.55b	16548.76 ± 286a
		TN	-	5717.51 ± 74.74c	7323.76 ± 83.37c	13074.26 ± 96.79c
	DCS	DC	-	8744.33 ± 118.62c	-	8744.33 ± 118.62e
		DR	1160.49 ± 28.29b	9599.25 ± 112.46a	-	10759.75 ± 110.65d
		DW	1518.52 ± 49a	9349.26 ± 93.07b	-	10884.47 ± 45.9d
		DN	-	8778.61 ± 93.48c	-	8778.61 ± 93.48e

## Discussion

### Effects of different cropping modes on soil microbial diversity

The microbial diversity is considered as an important indicator to assess the soil environment. The diversity of soil microbes is affected by soil nutrients, structure, pH, temperature, humidity, and ground cover [35–37]. It has been well documented that cover cropping has a significant impact on soil moisture and soil microclimate, leading to a significant change in soil microbial diversity and abundance [38, 39]. In the present study, triple multiple winter cropping increased the microbial community abundance index, diversity, and richness. The multiple cropping accelerates the soil carbon cycle and decomposition of SOM, which improve the overall soil fertility and microbial diversity [40]. Previously, different studies also noted that bacterial community structure and diversity are significantly changed in multiple cropping rice rotation as than the single cropping [41, 42].

Bacteria have higher activity in humid conditions [1, 2], and this study showed that multiple winter cropping significantly improved the abundance of the bacterial community, possibly by maintaining a higher soil water content. Moreover, the type of winter cover crops and their growth patterns also differently affect the structure and diversity of microbial communities [43]. These changes may result from the chemical characteristics of winter crops and plant-soil-biological interaction. For instance, leguminous plants (CMV) associate with nitrogen-fixing rhizobia and produce low C: N residues, which affect microbial nitrogen mineralization activity and soil nitrogen availability [44]. Besides this, combined return of winter crop straw and rice straw effectively increases SOM, and straw decomposition can increase the release of more available nutrients, thus providing rich carbon and nitrogen sources and resulting in an increase in soil bacterial community in paddy fields [45, 46].

The results indicated that multiple cropping also increased the soil nutrient concentration, and the difference in nutrient characteristics of farmlands shaped the difference in soil dominant bacterial genus [47, 48]. In this study, different winter multiple cropping increased the soil pH, TN, TP, and MBC contents in paddy fields (Table 6). The correlation between bacterial community diversity and abundance index in different winter multiple cropping patterns showed that (Table 6), soil AK, TK, and microbial species diversity were significantly correlated (*P* < 0.05). This shows that winter cropping provides more abundant nutrients and a higher relative humidity soil environment for microbial growth, thus changing the microbial community composition. Likewise, previous studies also proved the interaction between the co-existence of soil microbes and soil nutrient concentrations [49, 50]. We found that soil ammonium nitrogen was increased in multiple cropping, which might increase the competitive relationship between bacterial interactions, which consequently increased the bacterial community [51, 52]. The results of this study also showed that winter crop species and the rice rotational cropping pattern were two direct factors affecting soil bacterial diversity. Therefore, when using rotation to improve soil micro-ecology, both factors should be considered to play their best roles.

### Effects of different winter multiple cropping modes on soil microbial community structure

The results of the present study indicated that winter multiple cropping significantly affected the composition of microbial communities. The dominant populations in the soil samples of all winter multiple cropping treatments were *Proteobacter*,* Acidobacter*,* Nitrospirae*,* Chloroflexi*, and *Actinobacteria*. These results are similar to the findings of Liu et al. [1]; they found that three bacterial groups (*Nitrospirae*,* Chloroflexi* and *Actinobacteria*) were dominant populations in dry-land cropping [1]. Conversely, other authors found that *Proteobacteria* and *Actinobacteria* were the most dominant bacteria in alpine grassland, plateau orchard and dolomite karst [53, 54].

We found that the relative abundance of *Proteobacteria*,* Acidobacteria*, and *Actinomyceta* were significantly different in multiple cropping nodes as compared to their control treatments. In winter, straw mulching and straw returning effectively increases SOM and nutrient release and availability which provide a carbon and nutrient-rich environment for microbes, thereby increases microbial diversity and community structure [54]. Besides, rich organic matter and nitrate nitrogen under winter straw and rice straw returning provide abundant metabolic substrates for *Proteus*, which can effectively stimulate its rapid growth. Thus, the diversified crops induced changes in soil composition which increased the microbial diversity and community structures, which ensures better ecosystem functioning [55–58]. Moreover, the introduction of CMV to winter cropping system makes reasonable use of light, and heat sources, and it also reduces the fertilizer use and increases the SOM and soil nutrient concentration which in turn increases the overall soil microbial diversity and community structures [59, 60].

The results of cluster analysis indicate that the community structure of soil bacteria was changed after six years of winter multiple cropping (Fig. 3). TN and DN, treatments of winter multiple cropping system and two treatments of winter fallow control, were located in two clusters, indicating that different cropping systems with different winter crops changed the bacterial community structure. This may be due to the difference in crop root growth and soil nutrients released by different winter crops and rice rotation, which affects the growth and reproduction of microorganisms, and leads to changes in bacterial community and structure in soil [27, 61]. Moreover, winter crop species, soil properties, and agricultural management measures also have an impact on the soil microbial community structure of multiple cropping paddy fields. However, the interaction between winter crops, microorganisms and soil environment, and the contribution of various environmental factors to community structure needs further investigation.

### Effect of multiple cropping on yield of paddy fields

The results indicated that the annual yield of triple cropping system (TCS) was higher than the double rice cropping system (Table 7). In TCS annual yield of TC, TR, and TW was higher than the control treatment; similarly, in the DCS, the annual yield of DR and DW was higher than the control treatment (Table 7). The crop type and incorporation of straw significantly affect and change the microbial diversity and community structures [62, 63]. The addition of legume crops in multiple cropping and returning of straws and residues to the field increases the soil pH, SOM, and soil nitrogen availability which in turn increases the overall growth and yield of crops [64–66]. The winter cropping also increased the dry matter accumulation owing to the input of different winter crop stubbles and straws. Different straw inputs increase the soil microbial activities and increase the release of nutrients (Table 6), which therefore improved the growth and yield of rice [67]. Moreover, with an increase in cropping diversity, the community structure and diversity of microbes are also changed. In the present study, multiple cropping increased the diversity and abundance of microbes. The soils with higher SOC and ammonium nitrogen are considered to be rich in *Acidobacteria* members. In this study, multiple cropping increased the SOM and MBC, which increased the diversity and composition of the aforementioned and other bacteria, thus, resulting in a substantial increase in rice yield in multiple cropping as compared to mono-cropping [68, 69].

## Conclusion

Winter cropping in paddy field was proved beneficial to improve the fertility of the paddy field. Soil potassium and nitrogen contents were the key factors affecting the diversity and abundance of bacterial communities in different winter multiple cropping models. Hierarchical cluster analysis of soil bacterial communities showed that different cropping systems changed the population distribution of microorganisms. The dominant groups were *Proteus*,* Acidobacter*,* Nitrothyrobacter*, and *Campylobacter*, and the proportion of these four dominant groups was 80%. Multiple winter cropping increased the relative abundance of *Proteus*,* Acidobacter*, and *Actinomyces* and decreased the relative abundance of *Verrucomycetes* and *Archaea*. Green manure and rape planting in winter were beneficial to improve the soil fertility, thus improving soil bacterial diversity, bacterial community abundance and rice yield.

### Electronic supplementary material

Below is the link to the electronic supplementary material.

Supplementary Material 1

## Data Availability

All the data are available in the manuscript and with Correspondence authors.

## References

[1] Liu Z, Huang FY, LI JL, Zhang P, Yang BP, Ding RX, Nie JF, Jia ZK. Effects of farmland mulching patterns on soil microbial diversity and community structure in dryland. Acta Ecol Sinica. 2021;41(07):2750–60.

[2] Xue XM, Wang LP, Han XP, Chen R, Wang JZ. Effects of different tree disk mulching on soil microbial community structure and diversity in dwarfing rootstock apple orchard. Acta Ecol Sin. 2021;41(04):1528–36.

[3] Li Z, Tian D, Wang B, Wang J, Wang S, Chen HY, Xu X, Wang C, He N, Niu S. Microbes drive global soil nitrogen mineralization and availability. Global Change Biol. 2019;25(3):1078–88. 10.1111/gcb.14557 30589163

[4] Li Z, Zeng Z, Song Z, Wang F, Tian D, Mi W, Huang X, Wang J, Song L, Yang Z, Wang J. Vital roles of soil microbes in driving terrestrial nitrogen immobilization. Global Change Biol. 2021;27(9):1848–58. 10.1111/gcb.15552 33560594

[5] Guo Y, Wang YM, Wu P. Influence of long-term manure application in paddy soil on the functional diversity of microbial community. Chin J Appl Environ Biol. 2019;25(03):593–602.

[6] Nie Y, Wang M, Zhang W, Ni Z, Hashidoko Y, Shen W. Ammonium nitrogen content is a dominant predictor of bacterial community composition in an acidic forest soil with exogenous nitrogen enrichment. Sci Total Environ. 2018;624:407–15. 29262382 10.1016/j.scitotenv.2017.12.142

[7] Li L, Xi Y, Chen E, He L, Wang L, Xiao X, Tian W. Effects of tillage and green manure crop on composition and diversity of soil microbial community. J Ecol Rural Environ. 2018;34(04):342–8.

[8] Lin X, Shi H, Wu L, Cheng Y, Cai S, Huang S, He S, Huang Q, Zhang K. Effects of cultivation methods on soil microbial community structure and diversity in red paddy. Ecol Environ. 2020;29(11):2206–14.

[9] Jia PL, Feng HY, Li M. Soil microbial diversity of black soil under different land use patterns on northeast China. Trans Chin Soc Agric Eng. 2020;36(20):171–8.

[10] Ji L, Yang YC, Wang J. Relationship between soil phenolic acids and the soil microbial community under different land uses. Acta Ecol Sinica. 2019;39(18):6710–20.

[11] Tsiafouli MA, Thébault E, Sgardelis SP, Deruiter PC, VanderPutten WH, Birkhofer K. (2015). Intensive agriculture reduces soil biodiversity across Europe. Glob. Chang. Biol. (2015); 21, 973–985. 10.1111/gcb.12752 25242445

[12] Trinchera A, Migliore M, Warren RD, Ommeslag S, Debode J, Shanmugam S, Dane S, Babry J, Kivijarvi P, Kristensen HL, Lepse L. Can multi-cropping affect soil microbial stoichiometry and functional diversity, decreasing potential soil-borne pathogens? A study on European organic vegetable cropping systems. Front Plant Sci. 2022;13:952910. 36237499 10.3389/fpls.2022.952910 PMC9552534

[13] Williams A, Birt HW, Raghavendra A, Dennis PG. Cropping system diversification influences soil microbial diversity in subtropical dryland farming systems. Microb Ecol. 2023;85:1473–84. 35840682 10.1007/s00248-022-02074-w PMC10167104

[14] Li X, Jousset A, DeBoer W, Carrión VJ, Zhang T, Wang X, Kuramae EE. Legacy of land use history determines reprogramming of plant physiology by soil microbiome. ISME J. 2019;13:738–51. 30368524 10.1038/s41396-018-0300-0 PMC6461838

[15] Van-der-Bom FJT, Williams A, Bell MJ. Root architecture for improved resource capture: trade-offs in complex environments. J Exp Bot. 2020;71:5752–63. 32667996 10.1093/jxb/eraa324

[16] Benitez MS, Ewing PM, Osborne SL, Lehman RM. Rhizosphere microbial communities explain positive effects of diverse crop rotations on maize and soybean performance. Soil Biol Biochem. 2021;159:108309. 10.1016/j.soilbio.2021.108309

[17] Kramer C, Gleixner G. Soil organic matter in soil depth profiles: distinct carbon preferences of microbial groups during carbon transformation. Soil Biol Biochem. 2008;40:425–33. 10.1016/j.soilbio.2007.09.016

[18] Williams A, Kane DA, Ewing PM, Atwood LW, Jilling A, Li M, Lou Y, Davis AS, Grandy AS, Huerd SC, Hunter MC. Soil functional zone management: a vehicle for enhancing production and soil ecosystem services in row-crop agroecosystems. Front Plant Sci. 2016;7:65. 26904043 10.3389/fpls.2016.00065 PMC4743437

[19] Tian W, Wang L, Li Y, Zhuang KM, Li G, Zhang JB, Xiao XJ, Xi YG. Responses of microbial activity, abundance, and community in wheat soil after three years of heavy fertilization with manure-based compost and inorganic nitrogen. Agric Ecosyst Environ. 2015;213:219–27. 10.1016/j.agee.2015.08.009

[20] Zhang S, Wang Y, Sun L, Qiu C, Ding Y, Gu H, Wang L, Wang Z, Ding Z. Organic mulching positively regulates the soil microbial communities and ecosystem functions in tea plantation. BMC Microbiol. 2020;20:1–13. 32349665 10.1186/s12866-020-01794-8 PMC7191807

[21] Lan GY, Li YW, Wu ZX, Xie GS. Soil bacterial diversity impacted by conversion of secondary forest to rubber or eucalyptus plantations: a case study of Hainan Island, South China. Sci. 2017;63:87–93.

[22] Qian HF, Zhang M, Liu GF, Lu T, Qu Q, Du BB, Pan XL. Effects of soil residual plastic film on soil microbial community structure and fertility. Water Air Soil Pollut. 2018;229:261. 10.1007/s11270-018-3916-9

[23] Dong WY, Si PF, Liu E, Yan CR, Zhang Z, Zhang YQ. Influence of film mulching on soil microbial community in a rainfed region of northeastern China. Sci Rep. 2017;7:8468. 28814759 10.1038/s41598-017-08575-w PMC5559608

[24] Huang GQ. Green development of paddy field farming systems in the Yangtze River Economic Belt. Chin J Eco-Agri. 2020;28(01):1–7.

[25] Zhai MY, Xu XL, Jiang XS. A method on information extraction of winter fallow fields in middle and lower reaches of Yangtze River by remote sensing. (2012); 14(3), 389–97.

[26] Ye X, Liu H, Li Z, Wang Y, Wang Y, Wang H, Liu G. Effects of green manure continuous application on soil microbial biomass and enzyme activity. J Plant Nutr. 2014;37:498–508. 10.1080/01904167.2013.867978

[27] Bernard E, Larkin RP, Tavantzis S. Compost, rapeseed rotation, and bio-control agents significantly impact soil microbial communities in organic and conventional potato production systems. Appl Soil Ecol. 2012;52:29–41. 10.1016/j.apsoil.2011.10.002

[28] Lei B, Wang J, Yao H. Ecological and environmental benefits of planting green manure in paddy fields. Agriculture; 2022. p. 12020223.

[29] Pu J, Li Z, Tang H, Zhou G, Wei C, Dong W, Jin Z, He T. Response of soil microbial communities and rice yield to nitrogen reduction with green manure application in karst paddy areas. Front Microbiol. 2023;13:1070876. 36699610 10.3389/fmicb.2022.1070876 PMC9869043

[30] Tang H, Xiao X, Li C. Effects of winter cover crop and straw returning on the functional diversity of rhizosphere microbial on double-crop rice paddies. Agric Sci Technol. 2018;19:1–11.

[31] Zhang LC, Shao JH, Lin YQ, Kuang XL, Zhang HL, Qing C, Ma L, Yao BS. Influence of microbial diversity and activity of soil on the rice-rice-rape rotation. Ecol Environ. 2017;26(02):204–10.

[32] Wear EK, Wilbanks EG, Nelson CE, Carlson CA. Primer selection impacts specific population abundances but not community dynamics in a monthly time-series 16S rRNA gene amplicon analysis of coastal marine bacterioplankton. Environ Microbiol. 2018;20(8):2709–26. 29521439 10.1111/1462-2920.14091 PMC6175402

[33] Kim K, Kim HJ, Jeong DH, Huh JH, Jeon KS, Um Y. Correlation between soil bacterial community structure and soil properties in cultivation sites of 13-year-old wild-simulated ginseng (*Panax ginseng* CA Meyer). Appl Sci. 2021;11(3):937. 10.3390/app11030937

[34] Zhang J, Liu Q, Wang D, Zhang Z. Soil microbial community, soil quality, and productivity along a chronosequence of *Larix principis-rupprechtii* forests. Plants. 2023;12(16):2913. 37631125 10.3390/plants12162913 PMC10458017

[35] Vander-Heijden MGA, Wagg C. Soil microbial diversity and agro-ecosystem functioning. Plant Soil. 2013;363(1):1–5. 10.1007/s11104-012-1545-4

[36] Kennedy AC. Bacterial diversity in agro-ecosystems. Agricul Eco Environ. 1999;74(1):65–76. 10.1016/S0167-8809(99)00030-4

[37] Tang M, Li L, Wang X, You J, Li J, Chen X. Elevational is the main factor controlling the soil microbial community structure in alpine tundra of the Changbai Mountain. Sci Rep (2020); 10, 1–15. 10.1038/s41598-020-69441-w PMC7381615 32709903

[38] Tan G, Liu Y, Peng S, Yin H, Meng D, Tao J. Soil potentials to resist continuous cropping obstacle: three field cases. Environ Res. 2021;200:111319. 34052246 10.1016/j.envres.2021.111319

[39] Acosta-Martínez V, Burow G, Zobeck TM. Soil microbial communities and function in alternative systems to continuous cotton. Soil Sci Soc Amer J. 2010;74(4):1181–92. 10.2136/sssaj2008.0065

[40] Zheng H, Huang H, Zhang C, Li J. National-scale paddy-upland rotation in Northern China promotes sustainable development of cultivated land. Agric Water Manag. 2016;170:20–5. 10.1016/j.agwat.2016.01.009

[41] Li W, Liu M, Wu M, Jiang C, Kuzyakov Y, Gavrichkova O. Bacterial community succession in paddy soil depending on rice fertilization. Appl Soil Ecol. 2019;144:92–7. 10.1016/j.apsoil.2019.07.014

[42] Do TX, Vo TG, Rosling A, Alstrom S, Chai B, Hogberg N. Different crop rotation systems as drivers of change in soil bacterial community structure and yield of rice, Oryza sativa. Biol Fertil Soils. 2012;48:217–25. 10.1007/s00374-011-0618-5

[43] Bünemann EK, Bossio DA, Smithson PC. Microbial community composition and substrate use in a highly weathered soil as affected by crop rotation and P fertilization. Soil Biol Bioch. 2004;36(6):889–901. 10.1016/j.soilbio.2004.02.002

[44] Kumar K, Goh KM. In: Sparks, D.L, editor, Crop residues and management practices: effects on soil quality, soil nitrogen dynamics, crop yield, and nitrogen recovery. Advan. Agron. Acad. Press. (1999); 197–319.

[45] Zhou Y, Zhang Z, Fan R, Qian X, Lu X, Liu L. Carbon metabolism diversity characteristics of soil microbe affected by wheat straw incorporation pattern. J Ecol Rural Environ. 2017;33(10):913–20.

[46] Lu HF, Zheng JW, Yu XC, Zhou HM, Zheng JF, Zhang XH, Liu XY, Cheng K, Li IQ, Pan GX. Microbial community diversity and enzyme activity of red paddy soil under long-term combined inorganic-organic fertilization. J Plant Nutri Ferti. 2015;21(03):632–43.

[47] Shi Y, Delgado-Baquerizo M, Li Y, Yang Y, Zhu YG, Penuelas J. Abundance of kinless hubs within soil microbial networks are associated with high functional potential in agricultural ecosystems. Environ. Int. (2020); 142, 105869. 10.1016/j.envint.2020.105869 32593837

[48] Li M, Guo J, Ren T, Luo G, Shen Q, L. J. Crop rotation history constrains soil biodiversity and multifunctionality relationships. Agric Ecosyst Environ. 2021;319:107550. 10.1016/j.agee.2021.107550

[49] Mohapatra M, Yadav R, Rajput V, Dharne MS, Rastogi G. Metagenomic analysis reveals genetic insights on biogeochemical cycling, xenobiotic degradation, and stress resistance in mudflat microbiome. J Environ Manage (2021); 292. 10.1016/j.jenvman.2021.112738 34020306

[50] Yuan MM, Guo X, Wu L, Zhang Y, Xiao N, Ning D. Climate warming enhances microbial network complexity and stability. Nat Clim Chang (2021); 11, 343–U100.

[51] Ghoul M, Mitri S. The ecology and evolution of microbial competition. Trends Microbiol. 2016;24:833–45. 27546832 10.1016/j.tim.2016.06.011

[52] Wang S, Wang X, Han X, Deng Y. Higher precipitation strengthens the microbial interactions in semi-arid grassland soils. Glob Ecol Biogeogr. 2018;27:570–80. 10.1111/geb.12718

[53] Jin ZW, Zhong WH, Wu SS. Effect of vegetation on microbial communities in alpine grassland soils in Northwest Yunnan. Acta Microb Sinica. 2018;58(12):2174–85.

[54] Tang J, Li Y, He X. The diversity analysis of soil microbial community based on the high throughput sequencing under the dolomite karst rocky desertification environment. 2020; 38(05):20–8.

[55] Yachi S, Loreau M. Biodiversity and ecosystem productivity in a fluctuating environment: the insurance hypothesis. Proceed Natl Acad Sci. 1999;96:1463–8. 10.1073/pnas.96.4.1463 PMC15485 9990046

[56] Delgado-Baquerizo M, Reich PB, Trivedi C, Eldridge DJ, Abades S, Alfaro FD, Bastida F, Berhe AA, Cutler NA, Gallardo A. García-Velázquez L. Multiple elements of soil biodiversity drive ecosystem functions across biomes. Nat Ecol Evol. 2020;4(2):210–20. 32015427 10.1038/s41559-019-1084-y

[57] Jia J, Zhang J, Li Y, Koziol L, Podzikowski L, Delgado-Baquerizo M, Wang G, Zhang J. Relationships between soil biodiversity and multifunctionality in croplands depend on salinity and organic matter. Geoderma. 2023;429:116273. 10.1016/j.geoderma.2022.116273

[58] Jiao S, Lu Y, Wei G. Soil multitrophic network complexity enhances the link between biodiversity and multi-functionality in agricultural systems. Global Change Biol. (2022); 28; 140–153. 10.1111/gcb.15917 34610173

[59] Gao S, Zhou G, Cao W. Effects of milk vetch (*Astragalus sinicus*) as winter green manure on rice yield and rate of fertilizer application in rice paddies in South China. J Plant Nutr Fertil. 2020;26:2115–26.

[60] Zhou X, Liao Y, Lu Y, Rees R, Cao W, Nie J. Management of rice straw with relay cropping of Chinese milk vetch improved double -rice cropping system production in southern China. J Integr Agric. 2020;19:2103–15. 10.1016/S2095-3119(20)63206-3

[61] Yadvinder S, Bijay S, Ladha JK, Khind CS, Gupta RK, Meelu O, Pasuquin E. Long-term effects of organic inputs on yield and soil fertility in the rice–wheat rotation. Soil Sci Soc Amer J. 2004;68:845–53. 10.2136/sssaj2004.8450

[62] Chang F, Jia F, Lv R, Li Y, Wang Y, Jia Q. Soil bacterial communities reflect changes in soil properties during the tillage years of newly created farmland on the Loess Plateau. Appl Soil Ecol. 2021;16:103853. 10.1016/j.apsoil.2020.103853

[63] Tong AZ, Liu W, Liu Q, Xia GQ, Zhu JY. Diversity and composition of the Panax ginseng rhizosphere microbiome in various cultivation modesand ages. BMC Microbiol. 2021;21:1–13. 33419388 10.1186/s12866-020-02081-2 PMC7792351

[64] Zhong C, Liu Y, Xu X, Yang B, Aamer M, Zhang P. Paddy-upland rotation with Chinese milk vetch incorporation reduced the global warming potential and greenhouse gas emissions intensity of double rice cropping system. Environ Pollut. 2021;276:116696. 33744496 10.1016/j.envpol.2021.116696

[65] Alpha JM, Chen J, Zhang G. Effect of nitrogen fertilizer forms on growth, photosynthesis, and yield of rice under cadmium stress. J Plant Nutr. 2009;32:306–17. 10.1080/01904160802608635

[66] Wang ZH, Miao Yf, Li SX. Effect of ammonium and nitrate nitrogen fertilizers on wheat yield in relation to accumulated nitrate at different depths of soil in drylands of China. Field Crops Res. 2015;183:211–24. 10.1016/j.fcr.2015.07.019

[67] Song MY, Li ZP, Liu M, Liu MQ. Effects of mixtures of different organic materials on soil nutrient content and soil biochemical characteristics. Sci Agri Sinica. 2013;46:3594–603.

[68] Rannestad MM, Gessesse TA. Deforestation and subsequent cultivation of nutrient poor soils of miombo woodlands of tanzania: long term effect on maize yield and soil nutrients. Sustainability. 2020;12:4113. 10.3390/su12104113

[69] Chen T, Hu R, Zheng Z, Yang J, Fan H, Deng X, Yao W, Wang Q, Peng S, Li J. Soil bacterial community in the multiple cropping system increased grain yield within 40 cultivation years. Front Plant Sci. 2021;12:804527. 34987540 10.3389/fpls.2021.804527 PMC8721226

