# Supplementary material for: Multiple cropping effectively increases soil bacterial diversity, community abundance and soil fertility of paddy fields
Source: BMC Plant Biol. 2024 Jul 27;24:715. doi: 10.1186/s12870-024-05386-w (PMC11282777; doi:10.1186/s12870-024-05386-w)
Supplement: Supplementary file 1 — Supplementary Material 1 [file 12870_2024_5386_MOESM1_ESM.docx]

**Supplementary material**

**Multiple Cropping Effectively Increases Soil Bacterial Diversity, Community Abundance and Soil Fertility of Paddy Fields**

**Optimization sequence statistics**

The sample dilution curve is an important basis for characterizing whether the depth of high-throughput sequencing can cover all microbial groups in the sample. The results given in Figure 1 showed that number of OTUs in 24 soil samples increased rapidly first, then the slope of the dilution curve showed a downward trend, gradually changing to a slow rise. When the sequences number reached 4000, the curve generally tended to be stable, which indicated that the sequencing depth was reasonable. The data obtained by sequencing covered most of the bacterial species in the soil samples, which could greatly reflect the species composition in the samples.

**Table S1: Details of experimental treatments used in study**

| **Cropping**  **system** | **Pattern of multiple cropping** | **Abbreviation** | **Straw incorporation** |
| --- | --- | --- | --- |
| TCS | Chinese milk vetch-early rice-late rice | TC | All Chinese milk vetch and early rice straw were returned to the field; all late rice straw was returned as mulching |
|  | rape-early rice-late rice | TR | All rape and early rice straw were returned to the field;15 cm late rice straw stubble was returned to the field |
|  | wheat-early rice-late rice | TW | All wheat and early rice straw were returned to the field;15 cm late rice straw stubble was returned to the field |
|  | Winter fallow-early rice-late rice | TN | All early rice straw was returned to field; 15 cm late rice straw stubble was returned to the field |
| DCS | Chinese milk vetch-middle rice | DC | All Chinese milk vetch straw was returned to the field and all middle rice straw was returned as mulching. |
|  | Rape-middle rice | DR | All rape straw was returned to field; 15 cm middle rice straw stubble was returned to the field |
|  | Wheat-middle rice | DW | All wheat straw was returned to field，and 15 cm middle rice straw stubble was returned to the field |
|  | Winter fallow-middle rice | DN | 15 cm rice straw stubble was returned to the field |

Note: DCS and TCS mean double cropping system and triple cropping system, individually. In order to compare the effects of winter crops on a certain cropping system, in the comparative analysis, the winter fallow-middle rice model (DN) was used as the control of double winter multiple cropping system including TC, DR, DW, and the winter fallow-double cropping rice (TN) was used as the control of triple winter multiple cropping system including TC, TR, TW.

**Table S2: Details of sowing and harvesting dates and varieties used in winter crop planting**

| **Winter crops** | **Year** | **Variety** | **Sowing date** | **Harvest date** | **Planting pattern** |
| --- | --- | --- | --- | --- | --- |
| Chinese milk vetch | 2019 | Yujiang wide leaves | 2018/10/5 | 2019/5/3 | broadcast |
|  | 2020 | Yujiang wide leave | 2019/10/1 | 2020/5/2 | broadcast |
| Rape | 2019 | Ganyouza No.8 | 2018/11/5 | 2019/5/2 | hole seeding |
|  | 2020 | Ganyouza No.8 | 2019/10/28 | 2020/4/29 | hole seeding |
| Wheat | 2019 | Yangmai 23 | 2018/10/31 | 2019/5/3 | drill seeding |
|  | 2020 | Yangmai 23 | 2020/10/28 | 2020/5/3 | drill seeding |

**Table S3: Details of sowing and harvesting dates and varieties of ear and late rice**

| **Rice**  **season** | **Year** | **Variety** | **Transplanting date** | **Tillering**  **stage** | **Booting**  **stage** | **Full heading stage** | **Harvest**  **date** |
| --- | --- | --- | --- | --- | --- | --- | --- |
| Early rice | 2019 | Zhongzao 37 | 2019/5/5 | 2019/5/15 | 2019/6/10 | 2019/6/18 | 2019/7/15 |
|  | 2020 | Zhongzao 37 | 2020/5/8 | 2020/5/17 | 2020/6/10 | 2020/6/19 | 2020/7/14 |
| Middle rice | 2019 | Jingliangyouhuazhan | 2019/6/13 | 2019/6/20 | 2019/8/12 | 2019/8/18 | 2019/9/18 |
|  | 2020 | Jingliangyouhuazhan | 2020/6/13 | 2020/6/22 | 2020/8/11 | 2020/8/18 | 2020/9/26 |
| Late rice | 2019 | Rongyou huazhan | 2019/7/20 | 2019/7/25 | 2019/8/27 | 2019/9/6 | 2019/10/14 |
|  | 2020 | Rongyou huazhan | 2020/7/17 | 2020/7/25 | 2020/8/30 | 2020/9/10 | 2020/10/24 |
